# C/EBP β Mediates Endoplasmic Reticulum Stress Regulated Inflammatory Response and Extracellular Matrix Degradation in LPS-Stimulated Human Periodontal Ligament Cells

**DOI:** 10.3390/ijms17030385

**Published:** 2016-03-22

**Authors:** Yudi Bai, Yi Wei, Lian Wu, Jianhua Wei, Xiaojing Wang, Yuxiang Bai

**Affiliations:** 1 State Key Laboratory of Military Stomatology, Department of Pediatric Dentistry, School of Stomatology, the Fourth Military Medical University, Xi’an 710032, China; yiweiywy@tom.com (Y.W.); lianwulw@tom.com (L.W.); xiaojingwangxjw@163.com (X.W.); 2 Department of Oral and Maxillofacial Surgery, School of Stomatology, the Fourth Military Medical University, Xi’an 710032, China; jianhuaweijhw@163.com; 3 Department of Health Statistics, the Fourth Military Medical University, Xi’an 710033, China

**Keywords:** periodontitis, C/EBP β, endoplasmic reticulum stress, human periodontal ligament cells, *P. gingivalis* LPS

## Abstract

Periodontitis is an oral inflammatory disease that not only affects the integrity of local tooth-supporting tissues but also impacts systemic health. A compositional shift in oral microbiota has been considered as the main cause of periodontitis; however, the potential mechanism has not been fully defined. Herein, we investigated the role of CCAAT/enhancer-binding protein β (C/EBP β), a member of the C/EBP family of transcription factors, in human periodontal ligament cells (hPDLCs) exposed to *Porphyromonas gingivalis* (*P. gingivalis*) lipopolysaccharide (LPS). RT-PCR and Western blotting analysis showed that the expression of C/EBP β was significantly increased in hPDLCs stimulated with LPS stimuli. Overexpression of C/EBP β by the recombinant adenoviral vector pAd/C/EBP β markedly increased the expression of the pro-inflammatory cytokines IL-6 and IL-8, and matrix metalloproteinases (MMP)-8 and -9 in hPDLCs in response to LPS. Furthermore, the activation of endoplasmic reticulum (ER) stress was confirmed in LPS-stimulated hPDLCs by measuring the expression of the ER stress marker molecules protein kinase-like ER kinase (PERK), eIF2α, GRP78/Bip, and C/EBP homologous protein (CHOP). The ER stress inhibitor salubrinal repressed, but inducer tunicamycin enhanced, the production of IL-6, IL-8, MMP-8, and MMP-9 in hPDLCs. Additionally, ER stress inducer tunicamycin significantly increased the expression level of C/EBP β in hPDLCs. Blocking of C/EBP β by siRNA resulted in a significant decrease in the secretion of IL-6 and IL-8 and expression of MMP-8 and MMP-9 induced by tunicamycin treatment in hPDLCs. Taken together, ER stress appears to play a regulatory role in the inflammatory response and extracellular matrix (ECM) degradation in hPDLCs in response to LPS stimuli by activating C/EBP β expression. This enhances our understanding of human periodontitis pathology.

## 1. Introduction

Periodontitis is an oral inflammatory disease that affects the tooth-supporting tissues, including the alveolar bone, gingiva, parodontium, and cementum. A compositional shift in oral microbiota, especially *Porphyromonas gingivalis* (*P. gingivalis*), *Treponema denticola*, and *Tannerella forsythia*, has been considered as the main cause of periodontitis [1]. The accumulation of virulent oral bacteria on adjacent teeth results in the loss of connective tissue and causes bone loss, eventually affecting the integrity of the supporting structures. Dysfunction of the periodontium not only disrupts the local oral conditions but also affects systemic health [2,3]. Those patients with periodontitis face an increased risk of atherosclerosis, aspiration pneumonia, rheumatoid arthritis, and cancer. Hence, knowledge about the pathogenic mechanisms related to periodontal disease is urgently needed to improve the therapeutic strategies for human periodontitis.

Endoplasmic reticulum (ER) stress, a disequilibrium between the demand for ER function and the capacity of the ER, is caused by various factors, such as viral infection, genetic mutations, and other environmental factors. An elevated load of unfolded proteins in the ER induces the cells’ response to ER stress by activating the unfolded protein response (UPR) pathway to restore the normal function of ER [4]. It has been increasingly recognized that ER stress is associated with multiple biological processes, including inflammation, bone loss, cell apoptosis, and extracellular matrix degradation, and that it is involved in the progression of various diseases, such as inflammatory diseases and diabetes mellitus [4,5]. Upregulation of ER stress has also been observed in periodontitis. ER stress induced by *P. gingivalis* was proven to be involved in alveolar bone resorption in an experimental periodontitis mouse model [6]. Additionally, ER stress was reported to be activated by advanced glycation end products and to mediate inflammation in human periodontal ligament cells [7]. However, the precise mechanisms by which ER stress mediates inflammatory responses in periodontal disease remain unknown.

CCAAT/enhancer-binding protein β (C/EBP β) is a member of the family of transcription factors that contain a basic leucine zipper (bZIP) domain at the C terminus. The C/EBP members play roles in a wide range of cellular processes, such as cellular apoptosis, proliferation, adipocyte differentiation, carbohydrate metabolism and inflammation [8,9]. There is emerging evidence that C/EBP β is associated with ER stress and contributes to tumor cell death and migration [10,11]. Nevertheless, the interplay and potential roles of C/EBP β and ER stress in human periodontitis remain to be elucidated.

In the present study, we aimed to determine the expression and role of C/EBP β in periodontal ligament cells exposed to lipopolysaccharide (LPS). Furthermore, we investigated the role of ER stress, and addressed whether and how ER stress interacts with C/EBP β in periodontal ligament cells. The data obtained will enhance our understanding of human periodontitis pathology.

## 2. Results

### 2.1. C/EBP β Gene Expression Is Increased in Human Periodontal Ligament Cells (hPDLCs) Exposed to Lipopolysaccharide (LPS)

**Figure 1:**
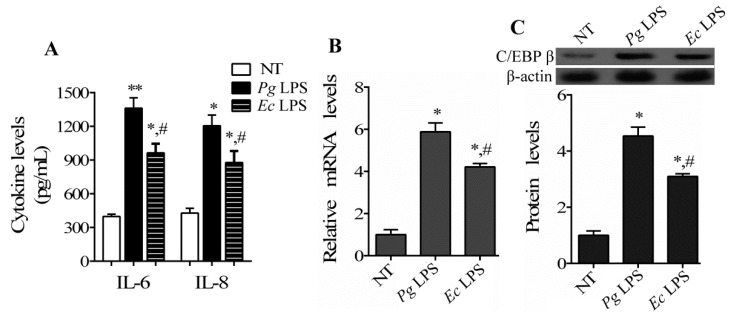
Expression of CCAAT/enhancer-binding protein β (C/EBP β) gene in human periodontal ligament cells (hPDLCs) stimulated by lipopolysaccharide (LPS). (**A**) hPDLCs were incubated with 1 μg/mL *Porphyromonas gingivalis* (*P. gingivalis*) LPS (*Pg* LPS) or *E. coli* LPS (*Ec* LPS) for 24 h. The levels of IL-6 and IL-8 in hPDLCs were measured using an ELISA kit; then, the mRNA (**B**) and protein (**C**) levels of C/EBP β in hPDLCs were analyzed by RT-PCR and Western blotting, respectively (* *p* < 0.05, ** *p* < 0.05 *vs.* not treated (NT); # *p* < 0.05 *vs. Pg* LPS).

To elucidate the role of C/EBP β in periodontal disease, we investigated the expression of the C/EBP β gene in hPDLCs exposed to LPS. The response of hPDLCs to stimulation with *P. gingivalis* LPS and *E. coli* LPS is shown in Figure 1A. Gene expression levels of pro-inflammatory cytokines IL-6 and IL-8 were markedly upregulated after stimulation with all stimuli. The increase in the levels of IL-6 and IL-8 in hPDLCs in response to stimulation with 1 μg/mL *P. gingivalis* LPS was higher than that in response to *E. coli* LPS (Figure 1A), indicating the suitability of LPS as a stimulant of hPDLCs. Further, the mRNA and protein expression levels of C/EBP β were significantly increased in hPDLCs stimulated with LPS in comparison with untreated (NT) cells. The increase in the expression level of C/EBP β in hPDLCs in response to *P. gingivalis* LPS was higher than *E. coli* LPS (Figure 1B,C). These data suggest that C/EBP β is aberrantly induced by LPS stimuli in hPDLCs. Thus, 1 μg/mL *P. gingivalis* LPS was used in subsequent experiments.

### 2.2. C/EBP β Induces the Production of Pro-Inflammatory Cytokines and Enhances the Activation of Matrix Metalloproteinases (MMPs) in hPDLCs Stimulated by LPS

**Figure 2:**
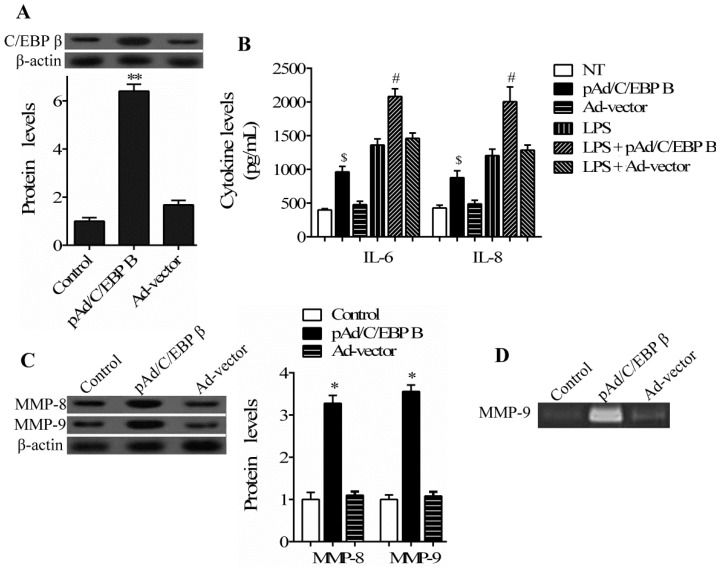
Effect of C/EBP β on production of pro-inflammatory cytokines and activation of matrix metalloproteinases (MMPs) in hPDLCs exposed to LPS. hPDLCs were transfected with a recombinant adenoviral vector pAd/C/EBP β or Ad-GFP control vector for 48 h, followed by stimulation with *P. gingivalis* LPS for another 24 h. (**A**) The protein level of C/EBP β in hPDLCs was determined by Western blotting; (**B**) The levels of IL-6 and IL-8 were analyzed by ELISA assay in hPDLCs transfected with adenoviral vector pAd/C/EBP β or Ad-vector in the presence or absence of LPS; (**C**) MMP-8 and MMP-9 levels in hPDLCs stimulated with LPS were analyzed by Western blotting assay; (**D**) gelatin zymography was performed to determine MMP-9 activity in hPDLCs stimulated with LPS. Untransfected hPDLCs were stimulated by *P. gingivalis* LPS and used as the control in (**A**,**C**,**D**). ($ *p* < 0.05 *vs.* NT; # *p* < 0.05 *vs.* LPS; * *p* < 0.05, ** *p* < 0.05 *vs.* control).

As an inflammatory response and extracellular matrix (ECM) degradation are the main pathological processes of periodontitis [12,13], we clarified the effect of C/EBP β on the production of pro-inflammatory cytokines and the activation of MMPs, major enzymes that are responsible for the degradation of ECM, in hPDLCs stimulated by LPS. Firstly, a recombinant adenoviral vector pAd/C/EBP β was constructed to overexpress C/EBP β in cultured hPDLCs. The levels of C/EBP β in cells treated with pAd/C/EBP β transduction and LPS stimulation was confirmed using a Western blotting assay (Figure 2A). Then the levels of IL-6 and IL-8 were evaluated in hPDLCs by ELISA. As seen in Figure 2B, C/EBP β overexpression increased the expression of cytokine IL-6 and IL-8 in hPDLCs stimulated by LPS or not with different extent, suggesting that C/EBP β is involved in the increase of IL-6 and IL-8 levels induced by LPS in hPDLCs.

Furthermore, we investigated the expression level of MMP-8 and MMP-9 in hPDLCs treated with pAd/C/EBP β and LPS by Western blotting assay. The results shown in Figure 2C indicate that MMP-8 and MMP-9 expression were detected in hPDLCs stimulated by LPS. Following pAd/C/EBP β transduction, the levels of MMP-8 and MMP-9 in hPDLCs were significantly increased. We then performed gelatin zymography assay to determine the effect of C/EBP β on MMP-9 activity. Compared to the control, the secretion of MMP-9 was significantly increased by C/EBP β overexpression (Figure 2D). Consistent with the results in Figure 2B, we also observed that C/EBP β overexpression increased the expression of MMP-8 and MMP-9 around 1.5-fold in unstimulated hPDLCs (data not shown), suggesting the regulatory role of transcription factors C/EBP β in hPDLCs in response to LPS stimuli.

### 2.3. Endoplasmic Reticulum (ER) Stress Regulates Inflammatory Response and Extracellular Matrix Degradation Induced by LPS in hPDLCs

**Figure 3:**
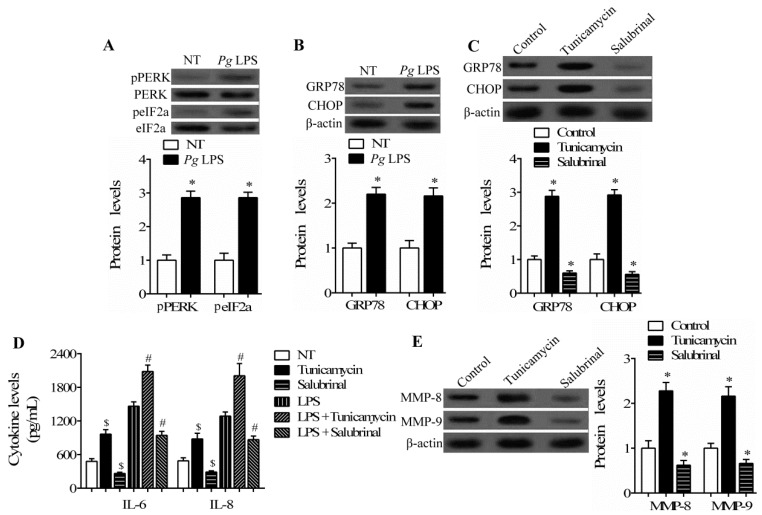
The role of ER stress induced by LPS in hPDLCs. hPDLCs were incubated with 1 μg/mL *P. gingivalis* LPS for 24 h. (**A**) The phosphorylation of protein kinase-like ER kinase (PERK), eIF2α and (**B**) the expression of GRP78/Bip and C/EBP homologous protein (CHOP) in hPDLCs were measured by Western blotting (* *p* < 0.05 *vs.* NT); (**C**) hPDLCs were pretreated with 5 μg/mL tunicamycin or 10 μM salubrinal for 2 h, followed by stimulation with *P. gingivalis* LPS for 24 h. The levels of GRP78/Bip and CHOP in hPDLCs were measured by Western blotting to confirm the efficiency of salubrinal and tunicamycin; (**D**) the levels of IL-6 and IL-8 were analyzed by ELISA in hPDLCs treated with salubrinal or tunicamycin in the presence or absence of LPS; and (**E**) Western blotting was used to measure the expression of MMP-8 and MMP-9 in hPDLCs stimulated with LPS. Untreated hPDLCs were stimulated by *P. gingivalis* LPS and used as the control. ($ *p* < 0.05 *vs.* NT; # *p* < 0.05 *vs.* LPS; * *p* < 0.05 *vs.* control).

Previous studies revealed that ER stress plays a crucial role in the inflammatory response and metabolism of ECM during normal physiological and pathological processes [2,12]. C/EBP β is associated with ER stress and contributes to cell death, migration, and inflammation [4,14]. Thereforewe attempted to determine the association between C/EBP β and ER stress in the progression of periodontitis. Firstly, activation of the ER stress signaling pathway induced by LPS stimuli was observed in hPDLCs by measuring the activation of ER stress-related marker molecules protein kinase-like ER kinase (PERK) and eIF2α. As seen in Figure 3A, the phosphorylation of both PERK and eIF2α was significantly upregulated. Next, we examined the expression of GRP78/Bip and CHOP, another two marker genes of ER stress, in hPDLCs. Western blotting assay indicated that GRP78/Bip and CHOP expression increased in hPDLCs after stimulation by LPS (Figure 3B), implying the activation of ER stress.

We then employed the ER stress inhibitor salubrinal (Calbiochem, San Diego, CA, USA) and inducer tunicamycin (Sigma, St. Louis, MO, USA) to elucidate the role of ER stress in LPS-treated hPDLCs. The inhibitory and inducing efficiency of salubrinal and tunicamycin were confirmed by detecting the expression of GRP78/Bip and CHOP (Figure 3C). Further analysis showed that salubrinal treatment successfully repressed, but tunicamycin increased, the secretion of IL-6 and IL-8 in hPDLCs stimulated by LPS or not, with different extents, suggesting that ER stress is involved in the increase of IL-6 and IL-8 levels induced by LPS in hPDLCs (Figure 3D). The expression of MMP-8 and MMP-9 were also decreased by salubrinal, but increased by tunicamycin, in hPDLCs stimulated by LPS (Figure 3E). Consistent with the results in Figure 3D, we also observed that the expression of MMP-8 and MMP-9 in unstimulated hPDLCs was only modestly affected by ER stress inhibition/induction (data not shown). These results suggest that LPS-induced ER stress plays a regulatory role in the inflammatory response and ECM degradation in hPDLCs in response to LPS stimuli.

### 2.4. C/EBP β Mediates the Regulatory Role of ER Stress in hPDLCs

**Figure 4:**
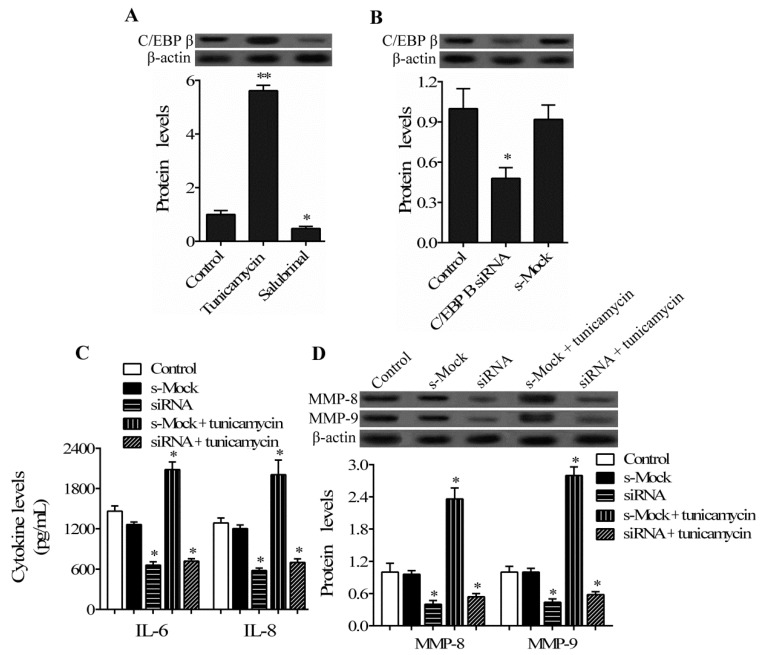
Interplay between C/EBP β and endoplasmic reticulum (ER) stress in hPDLCs. (**A**) hPDLCs were pretreated with 5 μg/mL tunicamycin or 10 μM salubrinal for 2 h, followed by stimulation with *P. gingivalis* LPS for 24 h. The expression of C/EBP β in hPDLCs was then analyzed by Western blotting; (**B**) hPDLCs were transfected with C/EBP β siRNA or non-targeting control siRNA (s-Mock) for 48 h, followed by stimulation with *P. gingivalis* LPS for another 24 h. The protein level of C/EBP β was determined by Western blotting to confirm the efficiency of siRNA; (**C**,**D**) C/EBP β siRNA-transfected hPDLCs were pretreated with tunicamycin, followed by stimulation with *P. gingivalis* LPS. The levels of IL-6 and IL-8 and the expression of MMP-8 and MMP-9 in hPDLCs were then analyzed by ELISA and Western blotting, respectively. Untransfected hPDLCs treated with LPS were used as the control. (* *p* < 0.05, ** *p* < 0.05 *vs.* control).

To further investigate the interplay between ER stress and C/EBP β in hPDLCs in response to LPS stimuli, the levels of C/EBP β were confirmed using Western blotting in hPDLCs treated with ER stress inducer tunicamycin or inhibitor salubrinal in the presence of LPS stimulation. As shown in Figure 4A, tunicamycin treatment significantly increased the expression level of C/EBP β in hPDLCs. Compared with untreated cells, however, C/EBP β expression was markedly downregulated in hPDLCs after incubation with the ER stress inhibitor salubrinal.

Furthermore, we introduced the C/EBP β siRNA to verify whether or not C/EBP β is responsible for the ER stress signaling pathway in hPDLCs. The blocking efficiency of C/EBP β siRNA was confirmed by Western blotting assay (Figure 4B). The results showed that C/EBP β siRNA transfection resulted in a significant decrease in the enhanced secretion of IL-6 and IL-8 and expression of MMP-8 and MMP-9 induced by tunicamycin treatment in hPDLCs (Figure 4C,D). Consistent with the results in Figure 2B and Figure 3D, we also observed that the expression of MMP-8 and MMP-9 in unstimulated hPDLCs was only modestly affected by C/EBP β/ER stress manipulation (data not shown). Taken together, these results provide evidence that LPS-induced ER stress in hPDLCs activates the expression of C/EBP β, which is involved in the regulation of ER stress in hPDLCs in response to LPS stimuli.

## 3. Discussion

Periodontitis is a chronic infectious disease that affects the integrity of tooth-supporting periodontium. Within the periodontium, the periodontal ligament is the key connecting tissue among teeth and other tooth-supporting tissues, including alveolar bone, gingiva, parodontium and cementum alveolar bone [2]. Periodontal ligament cells (PDLCs) are fibroblast-like cells that can express several ECM-related molecules. hPDLCs also exhibit increased pro-inflammatory mediators and cytokines production in response to bacterial infection [15]. Generally, periodontal health requires a maintainable microbiota homeostasis and immuno-inflammatory state in the periodontium, in which hPDLCs play crucial roles [16]. In periodontitis, the host immune homeostasis is dysregulated by oral bacteria [17,18]. Disruption of oral microorganisms has been considered the traditional cause of periodontitis. *P. gingivalis* is recognized as the major cause of periodontitis. Therefore, most studies have primarily used *P. gingivalis* as the model pathogen to stimulate hPDLCs *in vitro* when investigating the periodontal host immune response to microbial stimuli [19]. In the present study, *P. gingivalis* LPS was proven to stimulate hPDLCs to produce pro-inflammatory cytokines IL-6 and IL-8, and was used as a stimulus for hPDLCs in subsequent experiments.

C/EBP β, a member of the C/EBP family of transcription factors, regulates the expression of target genes that are involved in cellular proliferation, apoptosis, and migration. Accumulating evidence has demonstrated the role of C/EBP β in mediating adipocyte differentiation and neoplastic metabolism [20,21,22]. Increased C/EBP β expression in mice is capable of inducing hepatic steatosis that may lead to the occurrence of nonalcoholic steatohepatitis [23]. C/EBP β also induces MMP1/3 expression via the p38 mitogen-activated protein kinase (MAPK) pathway to mediate tumor necrosis factors (TNF)-α-induced cancer cell migration, contributing to the regulation of cancer metastasis [24]. In terms of inflammation, C/EBP β plays key roles in the control of inflammation and host immunity by regulating the functions of immunologic effector cells (macrophages and granulocytes) and target cells [8,9]. Zimmermann *et al.* recently reported that C/EBP β is involved in the regulation of serotonin transporter 5-HTT expression in macrophages in response to bacterial LPS stimuli [21]. Additionally, the C/EBP family of transcription factor also have regulatory roles in periodontal development and pathology. Savage *et al.* proved that p20C/EBP β, a negative C/EBP isoform, reduced alveolar bone mass and site-specific dentin dysplasia in transgenic mice [25]. C/EBPα, another member of the C/EBP family, was reported to regulate the lineage commitment of osteoclasts, which play a critical role in periodontal diseases [26]. Our data here show that the expression level of C/EBP β was significantly upregulated in hPDLCs after stimulation by LPS, and C/EBP β overexpression increased the secretion of pro-inflammatory cytokines and the activation of MMPs, which are major mediators of the pathological progression of periodontitis, suggesting the important role of the transcription factor C/EBP β in periodontal diseases.

Another approach we took to identify the role of C/EBP β was to elucidate the interplay between ER stress and C/EBP β in hPDLCs in response to LPS stimuli. ER stress is a self-adaptive and survival pathway of eukaryotic cells in response to nervous physiological states or stimuli. It is well known that ER stress is involved in multiple biological processes and various diseases, including periodontitis [12,27]. In mammalian cells, three ER-localized transmembrane proteins: double-stranded RNA-dependent protein kinase-like ER kinase (PERK), inositol-requiring 1 (IRE-1), and activating transcription factor 6 (ATF6), are primary initiators and transducers of ER stress signaling cascades. In inflammatory diseases, the PERK, IRE-1, and ATF6 pathways have been identified to play crucial roles in ER stress-mediated inflammatory response by regulating NF-κB and c-Jun N-terminal kinase (JNK) activation [4]. Emerging evidence has shown that C/EBP β is associated with ER stress and contributes to cell death, migration, and inflammation. Meir *et al.* reported that C/EBP-β regulates ER stress-induced apoptosis in mouse and human cells [14]. In addition, C/EBP-β knockdown moderates inflammation and lipid accumulation in the mouse model of nonalcoholic steatohepatitis [23]. Nevertheless, the interaction and potential roles of C/EBP β and ER stress in human periodontitis are not well defined. Herein, we determined that ER stress is induced by LPS and plays a regulatory role in the inflammatory response and ECM degradation in hPDLCs in response to LPS stimuli. Furthermore, C/EBP β was found to be activated by ER stress in hPDLCs after stimulation with LPS and, thus, might be involved in the regulation of ER stress in hPDLCs in response to LPS stimuli. These observations are consistent with a previous study demonstrating that the expression of human C/EBP β is activated by ER stress via the unfolded protein response element downstream of the coding gene [28]. However, our study is not without its limitations. How C/EBP β is involved in ER stress should be investigated in a subsequent study.

In summary, we have demonstrated the novel role of C/EBP β in hPDLCs exposed to LPS, and shown that ER stress induced by LPS plays a regulatory role in the inflammatory response and ECM degradation in hPDLCs in response to LPS stimuli by activating C/EBP β expression. This provides new insights into the pathology of human periodontitis.

## 4. Experimental Section

### 4.1. Cell Culture

Human periodontal ligament cells (hPDLCs) were obtained from healthy donors undergoing orthodontic treatment as described previously [19]. Briefly, after tooth extraction, the middle portion of root surfaces was scraped to obtain human periodontal ligament (hPDL) tissue. The PDL tissue was cut into small pieces and washed with Dulbecco’s modified Eagle medium (DMEM) (Sigma, St. Louis, MO, USA), followed by incubation in DMEM medium containing collagenase for 2 h at 37 °C. The isolated cells were then washed three times and cultured in DMEM, supplemented with 10% fetal bovine serum (FBS) (Invitrogen, Carlsbad, CA, USA) and 1% penicillin/streptomycin (Sigma) at 37 °C in a 5% CO_2_ atmosphere. Cells from passages 3–5 were used for subsequent experiments. Approval was obtained from the Ethics Committee of School of Stomatology of Fourth Military Medical University (24 September 2014, NO. 20140911). All subjects gave informed consent.

### 4.2. LPS Preparation and Stimulation

Ultrapure *P. gingivalis* LPS and *Escherichia coli* LPS were obtained from Invivogen (San Diego, CA, USA). The hPDL-derived cells were grown to approximately 80% confluence in 24-well plates and stimulated with 1 μg/mL *P. gingivalis* LPS or *E. coli* LPS for 24 h. Cells were then harvested for RNA and protein extraction.

### 4.3. Cell Transfection

The recombinant adenoviral vector pAd/C/EBP β was constructed. Briefly, full-length C/EBP β cDNA was amplified by RT-PCR from total RNA and subcloned into the pAdTrack-cytomegalovirus (CMV) vector. Then the recombinant pAd/C/EBP β vector was generated according to the manufacturer’s protocol. Adenoviruses without apparent cytotoxicity were used at 20 multiplicity of infection (moi) for 48 h for hPDLCs transduction. Adenovirus vector (Ad-GFP) encoding green fluorescent protein (GFP) gene was used as the control.

The siRNA targeting C/EBP β mRNA was synthesized by Takara (Takara, Dalian, China). hPDLCs were cultured in 96-well plates to approximately 80% confluence and transfected with C/EBP β siRNA or non-targeting negative control siRNA using Lipofectamine 2000 (Invitrogen) for 48 h. Cells were then stimulated with *P. gingivalis* LPS for another 24 h and harvested for subsequent experiments. The transfection efficacy was determined by Western blotting assays.

### 4.4. Quantitative RT-PCR

Trizol reagent (Sigma) was applied to isolate total RNA in hPDLCs. Expression of C/EBP β was detected using the CellAmp Direct RNA Prep kit (Takara) for qPCR and the Protein Analysis kit (Takara). The primers were as follows: C/EBP β: 5′-CAGCGCCGTCTTCTCCTC-3′ (forward) and 5′-CAATGAAACCCCCAACGAAAC-3′ (reverse); Glyceraldehyde-3-phosphate dehydrogenase (GAPDH): 5′-ACCACAGTCCATGCCATCAC-3′ (forward) and 5′-ACCACAGTCCATGCCATCAC-3′ (reverse). The reaction was performed as follows: 10 min 95 °C; 40 cycles of 1 min 95 °C, 2 min 63 °C, 1 min 72 °C; and 10 min 72 °C for final annealing. Cycle threshold (*C*t) values of GAPDH were used as the internal control.

### 4.5. Western Blotting

Cultured cells were lysed with lysis buffer (20 mmol/L 4-(2-hydroxyethyl)-1-piperazineethanesulfonic acid (HEPES), 5 mmol/L KCl, 25 mmol/L MgCl, 1% Triton X-100, and 0.5% phenylmethylsulfonyl fluoride (PMSF) to obtain total proteins. Equal amounts of proteins were separated by SDS-PAGE gels (Invitrogen), then electrophoretically transferred to polyvinylidene fluoride (PVDF) membrane. The blots were incubated with primary antibodies (mouse anti-C/EBP β 1:1000 dilution, Abcam, Cambridge, UK; rabbit anti-matrix metalloproteinases (MMP)-8, MMP-9 1:2000 dilution, rabbit anti-PERK, GRP78/Bip 1:3000, Abcam; mouse anti-eIF2α 1:2000, Abcam; mouse anti-CHOP 1:1000, (Pierce, Rockford, IL, USA); mouse anti-phospho-PERK, phospho-eIF2α 1:1000 dilution (Cell Signaling Technology, Danvers, MA, USA) and mouse anti-β-actin 1:3000 dilution (ABclonal, College Park, MD, USA)) overnight at 4 °C, and labeled with horseradish peroxidase-conjugated secondary antibodies for 1 h at room temperature. Protein bands were then detected using enhanced chemiluminescence (ECL, Amersham Pharmacia, Piscataway, NJ, USA) and measured by Image Quant software ev2003.03 (GE Healthcare, Pittsburgh, NJ, USA).

### 4.6. ELISA

The hPDL-derived cells were stimulated with 1 μg *P. gingivalis* LPS or *E. coli* LPS for 24 h at 37 °C. The treated cells were measured for the secretion of IL-6 and IL-8 using an ELISA kit (Boster, Wuhan, China) according to the manufacturer’s instructions.

### 4.7. Gelatin Zymography

MMP-9 activity was measured by gelatin zymography assay as previously described [29]. In brief, proteins (80 mg) were collected from cells and separated by SDS-PAGE containing 0.1% gelatin at 4 °C. Then the gels were washed and incubated in buffer (10 mM CaCl_2_, 50 mM Tris–HCl (pH 7.5)) at 37 °C. After staining and destaining, bands were analyzed using Image Quant software.

### 4.8. Statistical Analysis

The data are presented as mean ± SEM. Statistical differences were analyzed by Student’s *t* test using GraphPad Prism 6 (La Jolla, CA, USA). All experiments were performed at least three times. A value of *p* < 0.05 was considered to be statistically significant.
